# Supplementary material for: Tyrosine kinase inhibitor, masitinib, limits neuronal damage, as measured by serum neurofilament light chain concentration in a model of neuroimmune-driven neurodegenerative disease
Source: PLoS One. 2025 May 14;20(5):e0322199. doi: 10.1371/journal.pone.0322199 (PMC12077730; doi:10.1371/journal.pone.0322199)
Supplement: S1 Appendix — (PDF) [file pone.0322199.s001.pdf]

Supporting information files for 'Tyrosine kinase inhibitor, masitinib, limits neuronal damage, as measured by serum neurofilament light chain concentration, in a model of neuroimmune-driven neurodegenerative disease'

Table of Contents

BODY WEIGHT RAW DATA (TREATMENT PERIOD D0-D15) 2

RELATIVE CHANGE IN BODY WEIGHT STATISTICAL ANALYSIS 3

ROTAROD PERFORMANCE RAW DATA (TREATMENT PERIOD D0-D15) 4

ROTAROD RELATIVE PERFORMANCE STATISTICAL ANALYSIS (D0-D15) 5

GRIP STRENGTH RAW DATA (TREATMENT PERIOD D0-D15) 6

GRIP STRENGTH STATISTICAL ANALYSIS (D0-D15) 7

SERUM NfL RAW DATA AT D1 & D8 8

SERUM NfL RAW DATA AT D15 10

INTERFERON GAMMA (IFNγ) RAW DATA AT D15 11

TUMOR NECROSIS FACTOR ALPHA (TNFα) RAW DATA AT D15 12

HUMAN INTERLEUKIN-1 BETA (IL-1β) RAW DATA AT D15 13

INTERLEUKIN-33 (IL-33) RAW DATA AT D15 14

MACROPHAGE INFLAMMATORY PROTEIN-2 (MIP-2) RAW DATA AT D15 15

KERATINOCYTE CHEMOATTRACTANT/HUMAN GROWTH-REGULATED ONCOGENE (KC/GRO) RAW DATA AT D15 16

BODY WEIGHT RAW DATA (TREATMENT PERIOD D0-D15)

Group 1: Negative [non-EAE] control
Group 2: EAE [vehicle] control
Group 3: Masitinib 50 mg/kg/d
Group 4: Masitinib 100 mg/kg/d

Gp	mice ID	0	2	5	8	12	15
1	0	21.2	20.7	20.9	21.6	21.9	21.8
	1	19.7	20.0	20.1	19.9	20.7	20.7
	2	19.3	19.3	19.4	20.0	20.0	19.3
	4	19.7	19.7	19.5	19.7	20.2	20.2
	5	18.2	18.8	18.3	18.9	19.4	19.3
	6	18.2	18.8	18.4	18.6	20.0	19.4
	7	19.3	19.2	19.2	19.6	19.5	20.2
	10	18.4	18.0	18.8	18.7	18.9	19.4
	11	20.4	21.1	20.5	21.3	21.9	22.4
	12	22.0	21.4	22.4	23.0	23.2	23.7
	13	21.0	20.7	21.5	21.0	21.2	21.5
	14	20.6	20.6	20.8	20.9	22.7	22.4
	15	19.3	18.0	19.5	19.0	21.2	21.2
2	3	18.2	19.0	18.4	18.1	18.6	17.8
	16	20.4	20.7	18.0	17.8	17.5	18.3
	17	19.1	18.0	18.3	18.1	19.0	18.2
	23	20.5	20.0	17.8	16.2	18.2	18.1
	25	20.1	20.0	18.9	18.6	20.0	19.0
	30	22.1	22.2	22.0	21.4	21.3	20.6
	41	15.0	15.6	13.7	15.2		
	51	20.0	20.9	21.2	21.2	21.0	21.1
	54	17.0	16.9	15.2	17.1	17.6	17.4
	56	21.8	21.3	20.0	19.1	21.1	20.5
	66	19.6	18.0	17.5	18.6	18.7	18.9
	71	21.9	21.3	19.2	18.0	19.8	18.5
	73	17.6	17.3	17.6	18.4	19.2	18.8
3	22	18.1	18.2	18.8	19.2	18.4	18.2
	27	17.2	17.5	17.7	18.2	18.9	15.8
	31	19.6	19.5	18.2	17.2	18.2	17.3
	33	20.3	19.3	18.0	18.0	18.2	18.0
	34	21.1	19.8	18.5	18.6	19.2	18.8
	36	17.8	17.1	17.1	17.8	18.3	17.7
	40	18.0	17.7	18.0	19.4	19.4	20.7
	53	17.2	17.5	18.8	19.6	20.4	18.9
	61	21.2	20.3	20.0	20.6	21.3	20.7
	63	17.0	16.3	17.2	16.5	17.8	17.4
	64	20.0	19.5	17.9	18.7	19.2	18.6
	67	19.3	18.2	19.2	20.4	19.2	19.1
	70	22.9	22.6	23.0	22.0	23.2	22.2
4	20	18.2	18.0	17.7	18.6	18.8	18.0
	24	18.0	16.6	17.7	18.6	20.2	19.7
	26	18.2	17.7	18.8	17.9	18.2	18.3
	32	19.8	18.7	19.0	20.0	20.8	21.6
	37	18.5	18.7	18.5	18.7	20.2	20.1
	44	19.9	18.5	18.6	19.7	19.8	20.7
	45	18.2	17.1	16.9	17.2	18.7	17.4
	46	19.5	18.8	19.7	19.7	20.9	20.2
	52	20.1	18.7	18.6	18.6	20.2	18.0
	60	20.0	18.4	18.4	18.3	20.0	19.0
	62	21.3	21.0	20.3	19.4	20.1	18.0
	65	20.9	20.8	20.5	20.6	20.9	21.0
	72	19.4	18.6	19.2	19.3	19.3	18.2

One mouse (ID: 41) from the EAE control group (2) was euthanized at D8, as per ethical criteria.

RELATIVE CHANGE IN BODY WEIGHT STATISTICAL ANALYSIS

Group 1: Negative [non-EAE] control
Group 2: EAE [vehicle] control
Group 3: Masitinib 50 mg/kg/d
Group 4: Masitinib 100 mg/kg/d

Mean	0	2	5	8	12	15
- - - - -	1.000	0.997	1.008	1.019	1.053	1.055
Vehicle at - mg/kg/day; PO; Bid	1.000	0.993	0.939	0.944	0.977	0.957
Masitinib at 50 mg/kg/day; PO; Bid	1.000	0.976	0.974	0.991	1.012	0.978
Masitinib at 100 mg/kg/day; PO; Bid	1.000	0.959	0.969	0.980	1.026	0.995

SEM	0	2	5	8	12	15
- - - - -	0.000	0.008	0.003	0.005	0.009	0.008
Vehicle at - mg/kg/day; PO; Bid	0.000	0.011	0.017	0.023	0.020	0.019
Masitinib at 50 mg/kg/day; PO; Bid	0.000	0.008	0.018	0.023	0.022	0.022
Masitinib at 100 mg/kg/day; PO; Bid	0.000	0.008	0.010	0.012	0.013	0.021

RBW Statistical analysis (P vlaues)

		0	2	5	8	12	15
1 vs 2	- - - - - vs Masitinib at 50 mg/kg/day; PO; Bid x 14	1.0000	0.7779	0.0035	0.0083	0.0028	0.0009
1 vs 3	- - - - - vs Masitinib at 50 mg/kg/day; PO; Bid x 14	1.0000	0.0956	0.1510	0.3695	0.0612	0.0038
1 vs 4	- - - - - vs Masitinib at 100 mg/kg/day; PO; Bid x 14	1.0000	0.0052	0.0019	0.0066	0.0483	0.0293
2 vs 3	Vehicle at -mg/kg/day; PO; Bid x 14 vs Masitinib at 50 mg/kg/day; PO; Bid x 14	1.0000	0.2486	0.1370	0.1585	0.3014	0.6053
2 vs 4	Vehicle at -mg/kg/day; PO; Bid x 14 vs Masitinib at 100 mg/kg/day; PO; Bid x 14	1.0000	0.0293	0.1239	0.3560	0.0569	0.1739
3 vs 4	Masitinib at 50 mg/kg/day; PO; Bid x 14 vs Masitinib at 100 mg/kg/day; PO; Bid x 14	1.0000	0.1585	0.6261	0.8576	0.6261	0.4887

ns : no significativity
* : p < 0.05
** : p < 0.01
*** : p < 0.001

ROTAROD PERFORMANCE RAW DATA (TREATMENT PERIOD D0-D15)

Group 1: Negative [non-EAE] control
Group 2: EAE [vehicle] control
Group 3: Masitinib 50 mg/kg/d
Group 4: Masitinib 100 mg/kg/d

Group	ID	D0	D2	D5	D8	D12	D15
Group 1	0	223	182	138	166	178	149
	1	168	93	144	105	148	78
	2	106	97	136	124	82	113
	4	52	156	168	148	142	149
	5	176	125	209	189	161	229
	6	268	249	289	284	231	234
	7	186	273	282	261	216	220
	10	92	111	153	229	122	131
	11	111	139	92	107	94	124
	12	164	185	166	213	168	217
	13	243	119	205	173	169	176
	14	223	183	170	206	140	90
	15	160	146	153	187	99	119
Group 2	3	83	22	0	19	79	47
	16	122	123	0	0	1	0
	17	79	7	0	0	12	9
	23	163	118	0	0	22	20
	25	111	126	27	11	3	38
	30	201	150	135	16	23	8
	41	38	2	0	0	0	0
	51	151	127	149	103	121	103
	54	56	2	0	0	0	0
	56	123	136	158	82	66	123
	66	196	19	0	9	84	112
	71	74	104	17	5	0	3
	73	25	1	0	0	0	4
Group 3	22	163	8	0	2	1	2
	27	30	28	0	2	1	2
	31	144	102	0	10	1	1
	33	173	22	0	2	1	2
	34	147	116	0	2	35	41
	36	104	20	16	61	83	19
	40	18	1	0	0	18	35
	53	84	16	11	15	75	3
	61	111	15	0	25	51	4
	63	22	0	1	1	30	32
	64	173	126	1	9	2	9
	67	62	1	1	1	0	0
	70	74	101	75	26	51	14
Group 4	20	163	96	0	0	3	12
	24	20	1	0	0	14	25
	26	62	0	0	0	12	2
	32	20	0	0	0	1	3
	37	152	116	0	0	49	81
	44	75	26	0	8	32	52
	45	116	22	0	6	49	8
	46	101	68	41	63	66	36
	52	137	91	11	11	18	5
	60	166	31	0	9	28	63
	62	163	104	0	6	20	7
	65	93	83	66	55	35	52
	72	34	1	0	0	0	2

One mouse (ID: 41) from the EAE control group (2) was euthanized at D8, as per ethical criteria

ROTAROD RELATIVE PERFORMANCE STATISTICAL ANALYSIS (D0-D15)

Group 1: Negative [non-EAE] control
Group 2: EAE [vehicle] control
Group 3: Masitinib 50 mg/kg/d
Group 4: Masitinib 100 mg/kg/d

	Mean	-	2	5	8	12	15
1	- - - - -	1.000	1.092	1.218	1.268	1.020	1.089
2	Vehicle at - mg/kg/day; PO; Bid x 14	1.000	0.580	0.263	0.156	0.263	0.303
3	Masitinib at 50 mg/kg/day; PO; Bid x 14	1.000	0.407	0.105	0.125	0.423	0.329
4	Masitinib at 100 mg/kg/day; PO; Bid x 14	1.000	0.386	0.092	0.119	0.276	0.326

	SEM	-	2	5	8	12	15
1	- - - - -	0.000	0.177	0.187	0.184	0.153	0.174
2	Vehicle at - mg/kg/day; PO; Bid x 14	0.000	0.139	0.121	0.072	0.097	0.095
3	Masitinib at 50 mg/kg/day; PO; Bid x 14	0.000	0.123	0.077	0.048	0.132	0.173
4	Masitinib at 100 mg/kg/day; PO; Bid x 14	0.000	0.091	0.060	0.061	0.064	0.100

RRPc Statistical analysis (P vlaues)

		0	2	5	8	12	15
1 vs 2	- - - - - vs Masitinib at 50 mg/kg/day; PO; Bid x 14	1.0000	0.0545	0.0005	0.0000	0.0005	0.0004
1 vs 3	- - - - - vs Masitinib at 50 mg/kg/day; PO; Bid x 14	1.0000	0.0032	0.0001	0.0000	0.0111	0.0019
1 vs 4	- - - - - vs Masitinib at 100 mg/kg/day; PO; Bid x 14	1.0000	0.0004	0.0000	0.0000	0.0000	0.0004
2 vs 3	Vehicle at -mg/kg/day; PO; Bid x 14 vs Masitinib at 50 mg/kg/day; PO; Bid x 14	1.0000	0.3695	0.8175	0.5215	0.4628	0.4464
2 vs 4	Vehicle at -mg/kg/day; PO; Bid x 14 vs Masitinib at 100 mg/kg/day; PO; Bid x 14	1.0000	0.1910	0.4267	0.7779	0.4966	0.8704
3 vs 4	Masitinib at 50 mg/kg/day; PO; Bid x 14 vs Masitinib at 100 mg/kg/day; PO; Bid x 14	1.0000	0.7389	0.4267	0.3173	0.8375	0.1439

ns : no significativity
* : p < 0.05
** : p < 0.01
*** : p < 0.001

GRIP STRENGTH RAW DATA (TREATMENT PERIOD D0-D15)

Group 1: Negative [non-EAE] control
Group 2: EAE [vehicle] control
Group 3: Masitinib 50 mg/kg/d
Group 4: Masitinib 100 mg/kg/d

Group	ID	D0	D2	D5	D8	D12	D15
Group 1	0	250	259	265	253	263	230
	1	252	253	202	249	216	207
	2	185	228	225	224	211	230
	4	174	228	242	186	243	184
	5	205	221	221	160	201	214
	6	220	196	172	182	256	226
	7	229	208	177	182	194	230
	10	235	232	189	185	236	243
	11	286	210	226	206	232	213
	12	256	242	238	196	241	238
	13	226	229	254	229	211	246
	14	246	228	254	220	205	226
	15	217	215	226	184	225	224
Group 2	3	162	123	117	136	156	170
	16	210	175	92	71	112	144
	17	163	87	67	101	141	143
	23	216	176	99	97	149	154
	25	272	181	141	170	174	170
	30	211	269	152	148	175	156
	41	107	43	42	77		
	51	242	145	172	186	198	206
	54	155	54	39	55	73	70
	56	225	200	206	170	212	183
	66	203	126	100	130	127	151
	71	300	236	109	59	117	100
	73	108	91	119	105	126	132
Group 3	22	108	83	47	98	134	149
	27	183	134	108	144	188	174
	31	219	201	109	136	156	140
	33	213	115	77	85	127	131
	34	228	188	82	102	152	172
	36	94	92	132	146	155	162
	40	149	78	86	120	177	194
	53	126	88	108	159	157	154
	61	202	150	174	182	173	174
	63	131	90	104	118	181	177
	64	211	143	106	99	157	141
	67	120	109	82	148	160	147
	70	257	220	226	199	210	234
Group 4	20	188	88	88	100	136	172
	24	141	100	93	106	139	170
	26	131	104	83	112	177	169
	32	112	57	90	99	153	164
	37	225	140	136	134	178	182
	44	209	130	90	102	155	170
	45	149	105	104	142	162	133
	46	220	177	158	145	166	161
	52	189	120	104	133	183	137
	60	192	168	100	120	154	180
	62	263	192	127	155	169	199
	65	233	228	208	157	193	218
	72	130	90	118	138	182	185

One mouse (ID: 41) from the EAE control group (2) was euthanized at D8, as per ethical criteria.

GRIP STRENGTH STATISTICAL ANALYSIS (D0-D15)

Group 1: Negative [non-EAE] control
Group 2: EAE [vehicle] control
Group 3: Masitinib 50 mg/kg/d
Group 4: Masitinib 100 mg/kg/d

Mean		-	2	5	8	12	15
1	- - - - -	1.000	1.003	0.986	0.900	0.999	0.989
2	Vehicle at -mg/kg/day; PO; Bid x 14	1.000	0.721	0.576	0.613	0.745	0.759
3	Masitinib at 50 mg/kg/day; PO; Bid x 14	1.000	0.757	0.677	0.851	1.035	1.047
4	Masitinib at 100 mg/kg/day; PO; Bid x 14	1.000	0.704	0.643	0.721	0.957	0.992

SEM		-	2	5	8	12	15
1	- - - - -	-	0.040	0.054	0.040	0.045	0.035
2	Vehicle at -mg/kg/day; PO; Bid x 14	-	0.066	0.067	0.062	0.066	0.070
3	Masitinib at 50 mg/kg/day; PO; Bid x 14	-	0.038	0.080	0.095	0.091	0.096
4	Masitinib at 100 mg/kg/day; PO; Bid x 14	-	0.039	0.043	0.047	0.074	0.073

RGSc Statistical analysis (P vlaues)

		0	2	5	8	12	15
1 vs 2	- - - - - vs Masitinib at 50 mg/kg/day; PO; Bid x 14	1.0000	0.0008	0.0004	0.0005	0.0090	0.0055
1 vs 3	- - - - - vs Masitinib at 50 mg/kg/day; PO; Bid x 14	1.0000	0.0004	0.0061	0.5903	0.9387	0.9387
1 vs 4	- - - - - vs Masitinib at 100 mg/kg/day; PO; Bid x 14	1.0000	0.0001	0.0002	0.0096	0.2282	0.4267
2 vs 3	Vehicle at -mg/kg/day; PO; Bid x 14 vs Masitinib at 50 mg/kg/day; PO; Bid x 14	1.0000	0.4887	0.3695	0.0483	0.0339	0.0442
2 vs 4	Vehicle at -mg/kg/day; PO; Bid x 14 vs Masitinib at 100 mg/kg/day; PO; Bid x 14	1.0000	0.8980	0.2931	0.3428	0.0644	0.0257
3 vs 4	Masitinib at 50 mg/kg/day; PO; Bid x 14 vs Masitinib at 100 mg/kg/day; PO; Bid x 14	1.0000	0.3173	0.8576	0.3428	0.7005	0.8576

ns : no significativity
* : p < 0.05
** : p < 0.01
*** : p < 0.001

SERUM NfL RAW DATA AT D1 & D8

Human neurofilament Standard Range										
Conc (pg/ml)	Signal duplicate1	Signal duplicate2	Signal Mean	Adj. Sig. Mean	Signal CV	% Recovery	% Recovery Mean	Calc. Conc.	Calc. Conc. Mean	Calc. Conc. CV
50000.00	206937	212632	209785.00	209784.5	1.92	98.47	100.00	49235.66	49998.91	2.16
12500.00	57230	58899	58065.00	58064.5	2.03	98.50	100.01	12312.22	12501.08	2.14
3125.00	15816	14963	15390.00	15389.5	3.92	103.20	100.31	3224.88	3134.72	4.07
781.25	4059	3948	4004.00	4003.5	1.96	99.81	98.37	779.76	768.50	2.07
195.31	1173	1169	1171.00	1171	0.24	103.29	103.09	201.73	201.34	0.28
48.83	396	361	379.00	378.5	6.54	104.46	97.65	51.01	47.68	9.86
12.21	184	196	190.00	190	4.47	91.58	100.64	11.18	12.29	12.73
0.00	123	121	122.00	122	1.16		NaN	0.19	NaN	NaN

Group 1: Negative [non-EAE] control
Group 2: EAE [vehicle] control
Group 3: EAE Masitinib 50 mg/kg/d
Group 4: EAE Masitinib 100 mg/kg/d
BL: Baseline; D1: day 1; D8: day 8

	Sample	Dilution factor	Signal duplicate1	Signal duplicate2	Signal Mean	Adj. Sig. Mean	Signal CV	Calc. Conc.	Calc. Conc. Mean	Calc. Conc. CV	Relative quantif D8/meanD1	Mean pg/mL	CV pg/ml
		2.0	854	876	865	865	1.80	278.67	282.95	2.14		277.35	11.35
	BL	4.0	501	503	502	502	0.28	284.27	285.04	0.38			
		8.0	297	305	301	301	1.88	258.04	264.07	3.23			
		2.0	8019	8001	8010	8010	0.16	3183.87	3180.15	0.17	1.00	3213.74	35.91
**GROUP 1**	D1	4.0	4169	4187	4178	4178	0.30	3208.36	3215.68	0.32	1.00		
		8.0	2222	2182	2202	2202	1.28	3277.36	3245.41	1.39	1.00		
		2.0	4003	4036	4020	4019.5	0.58	1536.80	1543.49	0.61	0.48	1574.03	39.70
	D8	4.0	2134	2113	2124	2123.5	0.70	1568.42	1560.04	0.76	0.49		
		8.0	1159	1193	1176	1176	2.04	1591.86	1618.57	2.33	0.50		
		2.0	1315	1269	1292	1292	2.52	459.32	450.27	2.84		462.00	14.75
	BL	4.0	732	729	731	730.5	0.29	462.51	461.35	0.36			
		8.0	448	431	440	439.5	2.74	487.38	474.40	3.87			
		2.0	17997	17048	17523	17522.5	3.83	7375.11	7173.51	3.97	1.00	7349.55	213.98
**GROUP 2**	D1	4.0	9073	9329	9201	9201	1.97	7241.70	7348.09	2.05	1.00		
		8.0	4849	4853	4851	4851	0.06	7523.80	7527.06	0.06	1.00		
		2.0	58672	58656	58664	58664	0.02	25277.03	25273.41	0.02	3.44	25709.09	673.85
	D8	4.0	31182	29698	30440	30440	3.45	26108.71	25462.88	3.59	3.55		
		8.0	16135	16196	16166	16165.5	0.27	26339.31	26390.98	0.28	3.58		
		2.0	22335	22075	22205	22205	0.83	9228.33	9172.57	0.86	1.00	9413.92	207.84
	D1	4.0	11649	11803	11726	11726	0.93	9390.56	9455.06	0.96	1.00		
**GROUP 3**		8.0	6110	6142	6126	6126	0.37	9587.87	9614.14	0.39	1.00		
		2.0	44554	44040	44297	44297	0.82	18940.87	18826.67	0.86	2.01	19235.83	392.96
	D8	4.0	23093	23315	23204	23204	0.68	19107.56	19202.96	0.70	2.03		
		8.0	12187	12181	12184	12184	0.03	19682.89	19677.86	0.04	2.09		
		2.0	31573	31591	31582	31582	0.04	13224.78	13228.70	0.04	1.00	13483.81	237.23
	D1	4.0	16515	16569	16542	16542	0.23	13491.68	13514.57	0.24	1.00		
		8.0	8513	8699	8606	8606	1.53	13553.89	13708.16	1.59	1.00		
**GROUP 4**		2.0	43615	43671	43643	43643	0.09	18523.74	18536.17	0.09	1.37	18772.03	443.13
	D8	4.0	22152	22522	22337	22337	1.17	18299.65	18458.40	1.22	1.36		
		8.0	12030	11913	11972	11971.5	0.69	19419.59	19321.52	0.72	1.44		

SERUM NfL RAW DATA AT D15

Human neurofilament Standard Range								
Conc (pg/ml)	Signal duplicate1	Signal duplicate2	Mean	CV	% Recovery Mean	Calc. Conc.	Calc. Conc. Mean	Calc. Conc. CV
50000.00	212292.00	212421.00	212357.00	0.04	100.06	50013.84	50030.78	0.05
12500.00	60710.00	57898.00	59304.00	3.35	99.88	12802.07	12484.75	3.59
3125.00	16562.00	15614.00	16088.00	4.17	99.23	3198.29	3100.79	4.45
781.25	4656.00	4453.00	4555.00	3.15	102.17	817.57	798.19	3.43
195.31	1313.00	1279.00	1296.00	1.86	99.13	196.63	193.62	2.20
48.83	447.00	454.00	451.00	1.10	97.70	47.13	47.71	1.71
12.21	241.00	227.00	234.00	4.23	105.38	13.95	12.86	11.90
0.00	149.00	142.00	146.00	3.40	NaN	0.32	NaN	NaN

Group 2: EAE [vehicle] control
Group 3: Masitinib 50 mg/kg/d
Group 4: Masitinib 100 mg/kg/d

Sample	Dilution factor	Signal duplicate1	Signal duplicate2	Signal Mean	Signal CV	Calc. Conc.	Calc. Conc. Mean	Calc. Conc. CV	Samples Conc. Mean (pg/mL)	Samples Conc. Mean CV (pg/ml)	% decrease NfL masitinib vs control
Pool Group 2 D15	16.00	2201.00	2214.00	2208.00	0.42	5711.80	5730.84	0.47	5760.47	31.60	
	4.00	7949.00	7869.00	7909.00	0.72	5831.10	5799.57	0.77			
	8.00	4133.00	4143.00	4138.00	0.17	5743.41	5751.01	0.19			
Pool Group 3 D15	16.00	2119.00	2044.00	2082.00	2.55	5471.84	5362.34	2.89	5395.94	32.85	-6.33
	4.00	7376.00	7516.00	7446.00	1.33	5380.16	5435.16	1.43			
	8.00	4051.00	3749.00	3900.00	5.48	5618.93	5390.34	6.00			
Pool Group 4 D15	16.00	1672.00	1777.00	1725.00	4.31	4173.90	4325.51	4.96	4256.74	55.96	-26.10
	4.00	6004.00	5830.00	5917.00	2.08	4308.97	4241.52	2.25			
	8.00	3013.00	3209.00	3111.00	4.45	4056.75	4203.19	4.93			

INTERFERON GAMMA (IFNγ) RAW DATA AT D15

IFNγ Standard Range

IFNγ Conc.	Signal duplicate1	Signal duplicate2	Signal Mean	Mean CV	% Recovery Mean	Calc. Conc. (pg/mL)	Calc. Conc. Mean (pg/mL)	Calc. Conc. CV (pg/mL)
1030.00	1366200.00	1321302.00	1343751.00	2.36	99.41	1048.28	1023.96	3.36
257.50	449291.00	456394.00	452843.00	1.11	101.71	259.53	261.90	1.28
64.38	123357.00	130734.00	127046.00	4.11	99.77	62.22	64.22	4.42
16.09	33053.00	35805.00	34429.00	5.65	99.16	15.28	15.96	5.99
4.02	9105.00	9430.00	9268.00	2.48	98.60	3.89	3.97	2.64
1.01	2519.00	2730.00	2625.00	5.68	101.51	0.98	1.02	2.64
0.25	764.00	776.00	770.00	1.10	100.11	0.25	0.25	6.22
0.00	108.00	104.00	106.00	2.67	NaN	0.00	NaN	1.34

Group 2: EAE [vehicle] control
Group 3: Masitinib 50 mg/kg/d
Group 4: Masitinib 100 mg/kg/d

Sample	Dilution factor	Signal duplicate1	Signal duplicate2	Signal Mean	Signal CV	Calc. Conc.	Calc. Conc. Mean	Calc. Conc. CV
Pool Group 2 D15	16	621	622	622	0.11	3.08	3.09	0.14
	4	2069	2227	2148	5.20	3.14	3.28	5.75
	8	1049	1076	1063	1.80	2.91	2.95	2.10
Pool Group 3 D15	16	408	397	403	1.93	1.76	1.73	2.75
	4	1329	1340	1335	0.58	1.91	1.92	0.66
	8	695	771	733	7.33	1.78	1.90	9.00
Pool Group 4 D15	16	443	487	465	6.69	1.98	2.11	9.10
	4	1416	989	1203	25.11	2.06	1.71	28.88
	8	749	780	765	2.87	1.95	2.00	3.50

TUMOR NECROSIS FACTOR ALPHA (TNFα) RAW DATA AT D15

TNFα Standard Range

TNFα Conc.	Signal duplicate1	Signal duplicate2	Signal Mean	Mean CV	% Recovery Mean	Calc. Conc. (pg/mL)	Calc. Conc. Mean (pg/mL)	Calc. Conc. CV (pg/mL)
622.00	562830.00	540968.00	551899.00	2.80	100.55	638.90	625.41	3.05
155.50	149528.00	145431.00	147480.00	1.96	97.44	153.76	151.52	2.09
38.88	42247.00	41793.00	42020.00	0.76	102.69	40.15	39.92	0.81
9.72	11613.00	11080.00	11347.00	3.32	101.67	10.13	9.88	3.57
2.43	3139.00	3090.00	3115.00	1.11	98.59	2.42	2.40	1.26
0.61	1037.00	915.00	976.00	8.84	96.83	0.64	0.59	11.86
0.15	444.00	423.00	434.00	3.43	105.87	0.17	0.16	6.99
0.00	209.00	207.00	208.00	0.68	NaN	NaN	NaN	NaN

Group 2: EAE [vehicle] control
Group 3: Masitinib 50 mg/kg/d
Group 4: Masitinib 100 mg/kg/d

Sample	Dilution factor	Signal duplicate1	Signal duplicate2	Signal Mean	Signal CV	Calc. Conc.	Calc. Conc. Mean	Calc. Conc. CV
Pool Group 2 D15	16	1518	1509	1514	0.42	16.53	16.47	0.51
	4	5416	5478	5447	0.80	17.73	17.84	0.88
	8	2811	2817	2814	0.15	17.06	17.08	0.17
Pool Group 3 D15	16	1332	1256	1294	4.15	14.07	13.57	5.22
	4	3876	4008	3942	2.37	12.25	12.48	2.64
	8	2265	2489	2377	6.66	13.31	14.07	7.70
Pool Group 4 D15	16	1366	1496	1431	6.42	14.52	15.38	7.93
	4	4570	2975	3773	29.90	14.70	11.90	33.31
	8	2714	2502	2608	5.75	16.39	15.66	6.59

HUMAN INTERLEUKIN-1 BETA (IL-1β) RAW DATA AT D15

IL-1β Standard Range

IL-1β Conc.	Signal duplicate1	Signal duplicate2	Signal Mean	Mean CV	% Recovery Mean	Calc. Conc. (pg/mL)	Calc. Conc. Mean (pg/mL)	Calc. Conc. CV (pg/mL)
1740.00	695613.00	751694.00	723654.00	5.48	101.01	1661.91	1757.52	7.69
435.00	214073.00	245823.00	229948.00	9.76	96.76	387.44	420.89	11.24
108.75	69807.00	72909.00	71358.00	3.07	105.01	111.49	114.19	3.35
27.19	17740.00	19481.00	18611.00	6.61	98.29	25.38	26.72	7.12
6.80	5149.00	5273.00	5211.00	1.68	99.06	6.64	6.73	1.84
1.70	1531.00	1581.00	1556.00	2.27	101.74	1.70	1.73	2.67
0.42	516.00	521.00	519.00	0.68	98.81	0.42	0.42	1.01
0.00	135.00	159.00	147.00	11.54	NaN	NaN	NaN	NaN

Group 2: EAE [vehicle] control
Group 3: Masitinib 50 mg/kg/d
Group 4: Masitinib 100 mg/kg/d

Sample	Dilution factor	Signal duplicate1	Signal duplicate2	Signal Mean	Signal CV	Calc. Conc.	Calc. Conc. Mean	Calc. Conc. CV
Pool Group 2 D15	16	217	205	211	4.02	1.14	1.04	14.03
	4	393	403	398	1.78	1.08	1.11	2.99
	8	262	242	252	5.61	0.97	0.88	14.27
Pool Group 3 D15	16	175	176	176	0.40	0.43	0.44	2.62
	4	321	306	314	3.38	0.75	0.72	6.76
	8	240	264	252	6.73	0.77	0.88	17.12
Pool Group 4 D15	16	178	193	186	5.72	0.48	0.61	29.07
	4	318	333	326	3.26	0.74	0.77	6.30
	8	233	212	223	6.67	0.71	0.62	20.82

INTERLEUKIN-33 (IL-33) RAW DATA AT D15

IL-33 Standard Range

IL-33 Conc.	Signal duplicate1	Signal duplicate2	Signal Mean	Mean CV	% Recovery Mean	Calc. Conc. (pg/mL)	Calc. Conc. Mean (pg/mL)	Calc. Conc. CV (pg/mL)
3220.00	832506.00	743400.00	787953.00	8.00	101.76	3476.92	3276.74	8.64
805.00	213792.00	243876.00	228834.00	9.30	107.05	800.58	861.75	10.04
201.25	53935.00	59921.00	56928.00	7.44	95.18	180.65	191.55	8.05
50.31	15246.00	15668.00	15457.00	1.93	92.47	45.83	46.52	2.11
12.58	4510.00	5191.00	4851.00	9.93	103.29	11.97	12.99	11.08
3.14	1436.00	1406.00	1421.00	1.49	100.00	3.18	3.14	1.82
0.79	529.00	538.00	534.00	1.19	107.27	0.83	0.84	1.85
0.00	155.00	164.00	160.00	3.99	NaN	NaN	NaN	NaN

Group 2: EAE [vehicle] control
Group 3: Masitinib 50 mg/kg/d
Group 4: Masitinib 100 mg/kg/d

Sample	Dilution factor	Signal duplicate1	Signal duplicate2	Signal Mean	Signal CV	Calc. Conc.	Calc. Conc. Mean	Calc. Conc. CV
Pool Group 2 D15	16	296	304	300	1.89	4.50	4.64	4.41
	4	697	691	694	0.61	5.00	4.97	0.86
	8	410	432	421	3.70	4.36	4.57	6.47
Pool Group 3 D15	16	201	193	197	2.87	1.20	1.07	17.23
	4	251	239	245	3.46	0.72	0.67	10.98
	8	203	223	213	6.64	0.63	0.80	29.63
Pool Group 4 D15	16	198	233	216	11.48	1.10	1.69	49.26
	4	495	530	513	4.83	3.00	3.17	7.61
	8	319	298	309	4.81	2.67	2.48	10.90

MACROPHAGE INFLAMMATORY PROTEIN-2 (MIP-2) RAW DATA AT D15

MIP-2 Standard Range

MIP-2 Conc.	Signal duplicate1	Signal duplicate2	Signal Mean	Mean CV	% Recovery Mean	Calc. Conc. (pg/mL)	Calc. Conc. Mean (pg/mL)	Calc. Conc. CV (pg/mL)
692.00	1174171.00	1121862.00	1148017.00	3.22	100.26	710.68	693.77	3.45
173.00	319998.00	304736.00	312367.00	3.45	99.84	177.23	172.72	3.69
43.25	88123.00	87356.00	87740.00	0.62	102.97	44.74	44.54	0.66
10.81	22488.00	22345.00	22417.00	0.45	95.77	10.39	10.36	0.48
2.70	6750.00	6449.00	6600.00	3.23	102.75	2.85	2.78	3.49
0.68	1869.00	1679.00	1774.00	7.57	96.49	0.69	0.65	8.59
0.17	610.00	577.00	594.00	3.93	103.92	0.18	0.18	5.11
0.00	102.00	110.00	106.00	5.34	NaN	NaN	NaN	NaN

Group 2: EAE [vehicle] control
Group 3: Masitinib 50 mg/kg/d
Group 4: Masitinib 100 mg/kg/d

Sample	Dilution factor	Signal duplicate1	Signal duplicate2	Signal Mean	Signal CV	Calc. Conc.	Calc. Conc. Mean	Calc. Conc. CV
Pool Group 2 D15	16	1910	1916	1913	0.22	11.34	11.36	0.25
	4	3827	3685	3756	2.67	12.27	12.02	2.93
	8	7403	7387	7395	0.15	12.58	12.57	0.17
Pool Group 3 D15	16	1838	1749	1794	3.51	10.86	10.56	3.98
	4	3665	3659	3662	0.12	11.70	11.69	0.13
	8	7117	7390	7254	2.66	12.06	12.31	2.88
Pool Group 4 D15	16	1409	1371	1390	1.93	8.02	7.89	2.23
	4	2969	2918	2944	1.23	9.28	9.19	1.36
	8	6682	6764	6723	0.86	11.26	11.34	0.93

KERATINOCYTE CHEMOATTRACTANT/HUMAN GROWTH-REGULATED ONCOGENE (KC/GRO) RAW DATA AT D15

KC/GRO Standard Range

KC/GRO Conc.	Signal duplicate1	Signal duplicate2	Signal Mean	Mean CV	% Recovery Mean	Calc. Conc. (pg/mL)	Calc. Conc. Mean (pg/mL)	Calc. Conc. CV (pg/mL)
2320.00	1814958.00	1733792.00	1774375.00	3.23	98.77	2344.62	2291.41	3.28
580.00	487090.00	465555.00	476323.00	3.20	105.36	624.87	611.08	3.19
145.00	112702.00	115690.00	114196.00	1.85	101.62	145.43	147.35	1.84
36.25	26410.00	26547.00	26479.00	0.37	94.83	34.29	34.38	0.37
9.06	6990.00	6717.00	6854.00	2.82	97.56	9.02	8.84	2.86
2.27	1865.00	1807.00	1836.00	2.23	98.97	2.28	2.24	2.42
0.57	603.00	618.00	611.00	1.74	108.24	0.60	0.61	2.31
0.00	160.00	148.00	154.00	5.51	NaN	0.01	NaN	NaN

Group 2: EAE [vehicle] control
Group 3: Masitinib 50 mg/kg/d
Group 4: Masitinib 100 mg/kg/d

Sample	Dilution factor	Signal duplicate1	Signal duplicate2	Signal Mean	Signal CV	Calc. Conc.	Calc. Conc. Mean	Calc. Conc. CV
Pool Group 2 D15	16	2918	2903	2911	0.36	58.75	58.59	0.38
	4	11823	11803	11813	0.12	61.33	61.28	0.12
	8	5823	5632	5728	2.36	59.93	58.92	2.40
Pool Group 3 D15	16	2806	2875	2841	1.72	56.39	57.11	1.80
	4	12288	12254	12271	0.20	63.76	63.67	0.20
	8	5848	5968	5908	1.44	60.19	60.82	1.46
Pool Group 4 D15	16	2418	2528	2473	3.15	48.19	49.35	3.33
	4	10953	7353	9153	27.81	56.79	47.39	28.08
	8	4591	4953	4772	5.36	46.99	48.89	5.50
